# Gut microbiota profile in adults with abdominal obesity and non-abdominal obesity: links to body composition and macronutrient intake in Semarang, Indonesia—a brief research report

**DOI:** 10.3389/fnut.2025.1648575

**Published:** 2025-09-08

**Authors:** Farhan Syafiq Fadhillah, Fariz Budi Arafat, Nani Maharani, Jihan Fadhilah, Hainun Zariyah, Mochamad Ali Sobirin, Hiroyuki Tsutsui, Adi Wibowo, Achmad Zulfa Juniarto, Adriyan Pramono

**Affiliations:** ^1^Department of Nutrition Science, Faculty of Medicine, Diponegoro University, Semarang, Indonesia; ^2^Department of Statistics, Faculty of Sciences and Mathematics, Diponegoro University, Semarang, Indonesia; ^3^Department of Pharmacology and Therapy, Faculty of Medicine, Diponegoro University, Semarang, Indonesia; ^4^Department of Cardiology and Vascular Medicine, Faculty of Medicine, Diponegoro University, Semarang, Indonesia; ^5^Department of Cardiovascular Medicine, Faculty of Medical Sciences, Kyushu University, Fukuoka, Japan; ^6^Department of Informatics, Faculty of Sciences and Mathematics, Diponegoro University, Semarang, Indonesia; ^7^Center of Biomedical Research, Diponegoro University, Semarang, Indonesia; ^8^Center of Nutrition Research, Diponegoro University, Semarang, Indonesia

**Keywords:** gut microbiota, abdominal obesity, microbial diversity, adults, dietary intake

## Abstract

**Background:**

Abdominal obesity is a significant risk factor for metabolic syndrome and cardiovascular diseases, with increasing evidence highlighting the role of gut microbiota in its development. In Indonesia, where 23.4% of adults are obese, few studies have examined the gut microbiota in relation to abdominal obesity, particularly in the context of unique dietary patterns. This pilot study investigated the gut microbiota composition in adults with abdominal obesity in Semarang, Indonesia, and its associations with body composition and macronutrient intake.

**Methods:**

This cross-sectional study was conducted in Semarang and included 46 adults aged 20–50 years, categorized by abdominal obesity status (22 with abdominal obesity and 24 without). Anthropometric measurements, body composition, and dietary intake were assessed. Gut microbiota profiles were analyzed using 16S rRNA gene sequencing of fecal samples.

**Findings:**

In the Semarang population, individuals with abdominal obesity had higher visceral fat (12.32 ± 3.44% vs. 6.96 ± 2.91%) compared to those without abdominal obesity. *Prevotella_9 copri* was positively associated with visceral fat (r = 0.206, *p* = 0.169), a finding that differs from studies conducted outside Indonesia, potentially showing the uniqueness of the profile.

**Conclusion:**

The correlation of *Prevotella_9 copri* in subjects from Semarang, Indonesia, differs from findings in other studies, providing a potential unique gut microbiota profile in the Indonesian population and providing a platform for future studies to clarify these hypotheses. Larger longitudinal studies are needed to validate these findings and establish causality.

## Graphical abstract

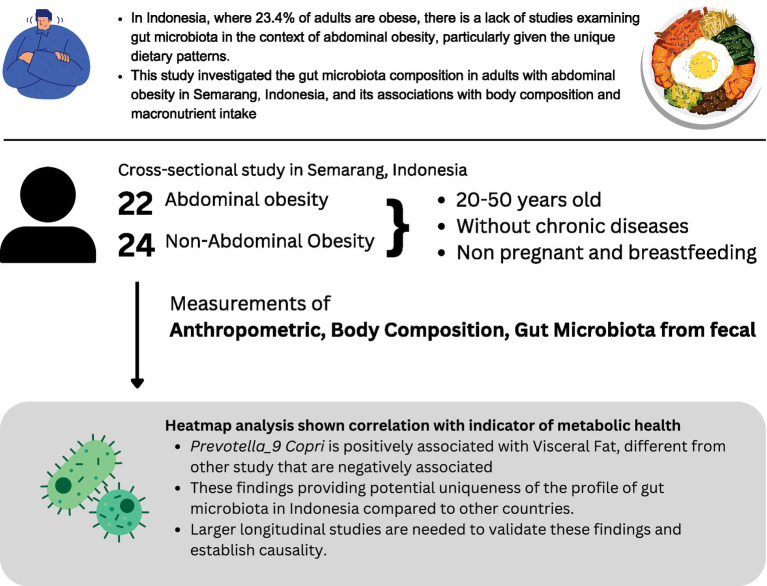

## 1 Introduction

Obesity affected 16% of the global adult population in 2024, with developing countries such as Indonesia currently experiencing rising rates; 23.4% of Indonesian adults were obese in 2023, up from 21.8% in 2018 (1, 2). Jakarta, the capital region, has a prevalence of abdominal obesity of 31.8%. There are some interesting findings where the coastal districts have a lower prevalence (for example, in the Nusa Tenggara Timur Province, where majority of the population lives in the rural areas, the prevalence is 13.3%) (2–5). Indonesia has distinct geographical characteristics, from coastal to non-coastal, with one study showing an improvement in the health quality in the coastal area. A study from Geiger SJ et al. shows better reported self-health in population near the coastal area (6). However, the study has yet to clarify whether living in coastal area is associated with lower body mass index (BMI) and/or reduced obesity prevalence. While rapid urbanization, nutritional transition, and lifestyle changes may have contributed to these trends, other factors may also explain the difference between abdominal obesity and other factors, such as gut microbiota where different dietary intake can alter the gut microbiota composition, producing different nutritional metabolism (7), possibly explaining the uniqueness of Indonesian population, where predominantly coastal living is associated with lower BMI and overall lower abdominal obesity (8). With that perspective, the study that overviews the profile of gut microbiota in adults with abdominal obesity and comparing with non-abdominal obesity in Indonesia is necessary, given the diversity of dietary intake in urban and rural areas of Indonesia (9).

BMI may not fully capture health risks from fat distribution; abdominal circumference better indicates visceral fat accumulation. Abdominal obesity’s strong link to visceral fat and metabolic complications has prompted microbiota investigations. Visceral fat, more than subcutaneous fat, correlates with metabolic syndrome, insulin resistance, and cardiovascular disease (4). Recognizing these associations is critical for public health and clinical practice, as understanding fat distribution patterns can improve early interventions and reduce obesity complications. Additionally, research indicates that gut microbiota significantly influences metabolic health. Individuals with altered microbial communities show metabolic changes with increased calorie absorption and adiposity, suggesting the microbiome’s potential as a metabolic biomarker and therapeutic target (10, 11). Differences in locations, such as coastal and non-coastal areas, could alter human gut microbiota through variations in dietary intake. Fıçıcılar’s study in Samsun, Turkey, showed higher fish intake in coastal areas, while Bratlie et al.’s research in Norway found that fish consumption was correlated with higher Firmicutes and lower Bacteroidetes compared to controls (12, 13).

To bridge these insights, this pilot study integrates abdominal circumference measures with gut microbiota profiles, body composition, and macronutrient intake in Indonesian adults with abdominal obesity and non-abdominal obesity, conducted by a cross-sectional study in Semarang, Indonesia, exploring the potential idea to benefit the population, researchers, and policymakers in precision nutrition.

## 2 Method

### 2.1 Study design

This cross-sectional study was conducted in northern Semarang, Indonesia, from June to December 2024, to examine the profile of the gut microbiota in adults with abdominal obesity and non-abdominal obesity. Semarang was selected for its rapid urbanization and evolving dietary patterns that reflect transitions in emerging economies (14). Data were collected from local community centers and health facilities across several sites. To minimize potential sources of bias, the data collection was conducted on the same day as fecal sampling, reducing the chance of fabricated results such as having dietary intake before or after the fecal collection. The study protocol was reviewed and approved by the Medical Ethics Committee of the Faculty of Medicine, Diponegoro University (322/EC/KEPK/FK-UNDIP/VI/2024).

### 2.2 Participants

The study enrolled adults aged 20–50 years residing in the northern part of Semarang, Indonesia, who were categorized based on their abdominal obesity status, with abdominal obesity defined as waist circumference of ≥90 cm (men) or ≥80 cm (women), according to the Indonesian cutoff (15). Exclusion criteria were chronic metabolic diseases, pregnancy, or breastfeeding. Participants were screened through questionnaires and clinical assessments. Recruitment occurred via community outreach, advertisements, and primary care providers. All participants provided informed consent per Declaration of Helsinki (16). This study enrolled a total of 46 participants: with 22 in abdominal obesity and 24 in non-abdominal obesity group. The sample size was limited by low participation during the one-month recruitment period in June and by resource constraints. The limited recruitment duration may introduce participation bias; however, as most participants were recruited from two clinical centers and given the nature of this pilot study, participation bias was not considered.

### 2.3 Study variables and measurements

#### 2.3.1 Anthropometric and vital sign measurements

Participants body weight and height were measured using calibrated digital scales and stadiometers. These values were used to calculate BMI as kg/m^2^. In addition, waist and hip circumferences were measured at standardized anatomical landmarks using non-stretchable tape. Waist circumference was emphasized as a critical measure because it reflects visceral fat accumulation, which is strongly associated with metabolic risk factors (17). Additionally, we measured the systole and diastole of the participants blood pressure.

#### 2.3.2 Body composition analysis

Body composition parameters, including fat mass, muscle mass, and visceral fat index, were evaluated using bioelectrical impedance analysis (BIA) (Tanita, Tokyo, Japan). BIA was selected because it is noninvasive, rapid, and cost-effective, with proven reliability compared to techniques such as dual-energy X-ray absorptiometry (DXA) (18).

#### 2.3.3 Dietary intake

Macronutrient and energy intakes were assessed using a validated semi-quantitative food frequency questionnaire (SQ-FFQ) specifically designed for the Indonesian population. The SQ-FFQ was pre-tested for reliability and validity in similar cohorts to capture the frequency and portion sizes of commonly consumed foods (19), and it is chosen instead of other tools such as diet quality questionnaire (DQQ) because of the non-specific and generalized nature of DQQ itself (20).

#### 2.3.4 Fecal DNA isolation and sequencing

Fecal samples were collected in sterile containers at −20°C during transport and processed at the laboratory. DNA was extracted from 200 mg fecal matter using QIAamp PowerFecal Pro DNA Kit (Qiagen, Hilden, Germany) per manufacturer’s protocol. DNA quality and yield were verified by spectrophotometry and gel electrophoresis. The V4 region of bacterial 16S rRNA gene was sequenced using Illumina MiSeq platform (Illumina, San Diego, CA, USA). Studies have validated this method for characterizing gut microbial communities (21).

### 2.4 Statistical methods

Data analysis was performed using R programming language (version 4.5.0; R Core Team, Vienna, Austria), with microbiome data processed using the phyloseq package (version 1.51.0). Baseline data were summarized using descriptive statistics. Groups defined by abdominal circumference were compared using the Mann–Whitney U test for alpha diversity, while beta diversity was assessed using Bray–Curtis dissimilarity calculated with the vegan package (v2.6–10) in R. Non-metric multidimensional scaling (NMDS) and principal coordinates analysis (PCoA) were used to visualize beta diversity patterns. Group-level differences were statistically evaluated using PERMANOVA. The taxa abundance was analyzed using QIIME2 (QIIME Development Team, Tucson, AZ, USA) and DESeq2 (22). Spearman rank correlation was used to assess associations between gut microbiota profiles and clinical parameters. To account for multiple comparisons, *p*-values were adjusted using the Benjamini–Hochberg procedure (false discovery rate, FDR) (23). The Spearman correlation analysis showed species with ≥80% abundance best visualized gut microbiota associations with biomarkers. A heatmap illustrated these correlations, enabling the interpretation of microbial patterns and inter-sample variability, as shown in other studies (24).

## 3 Results

### 3.1 Participants

**Figure 1:**
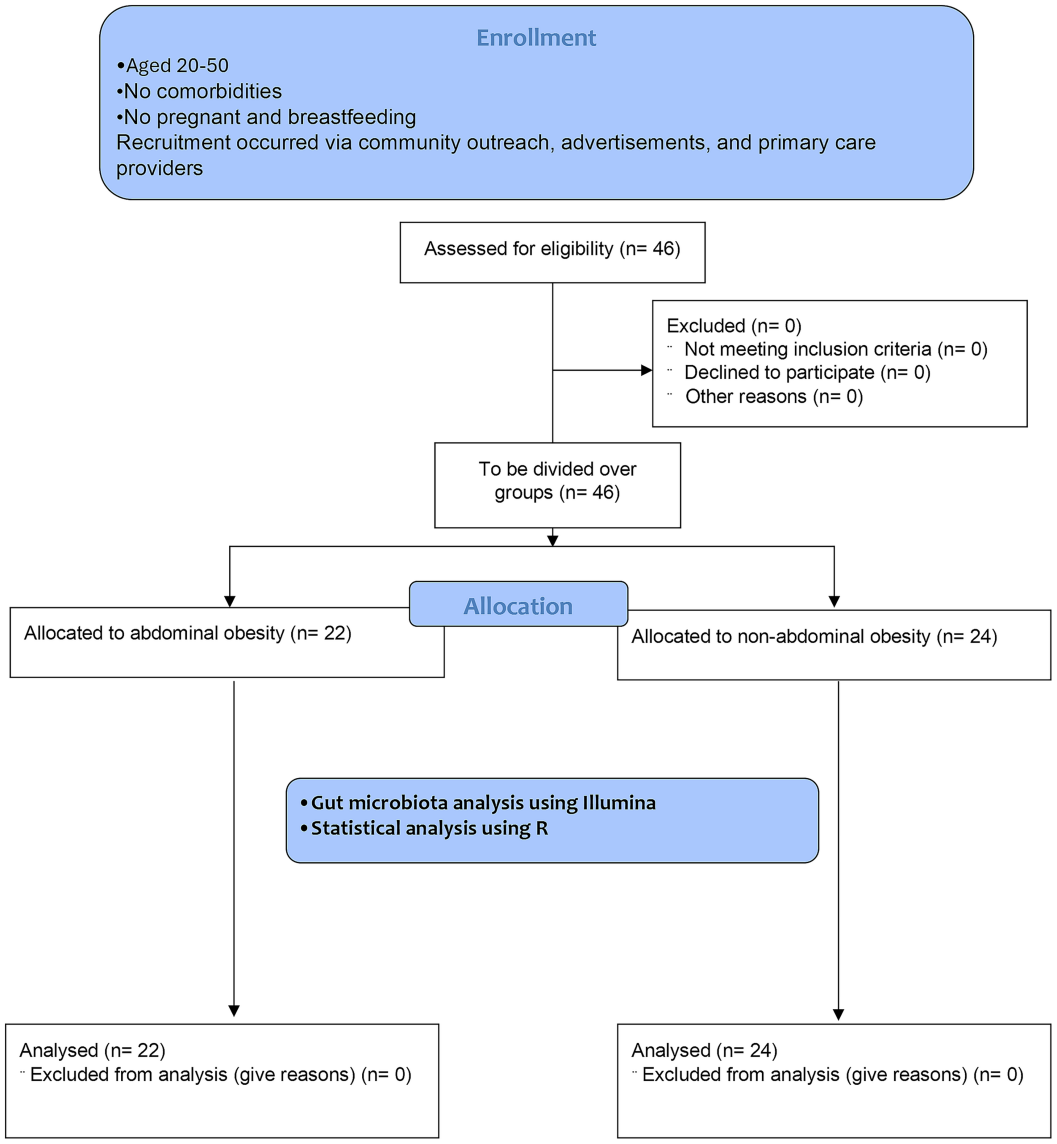
CONSORT 2010 flow diagram.

A total of 46 participants were recruited and categorized into two groups based on abdominal obesity status: 22 individuals with abdominal obesity and 14 individuals with a normal abdominal profile, with the flowchart available in Figure 1. In this study, there were clear differences between people with abdominal obesity and those with non-abdominal obesity, particularly the abdominal obesity group had a higher BMI (29.71 ± 5.34 kg/m^2^ vs. 23.32 ± 4.20 kg/m^2^), larger waist circumference (98.83 ± 11.11 cm vs. 81.37 ± 11.30 cm), and higher visceral fat (12.32 ± 3.44% vs. 6.96 ± 2.91%) compared to the normal obesity group. Full details are available in Table 1.

**Table 1:** Characteristics of individuals.

Subject characteristics	*p*-Value^a^	Abdominal obesity (*n* = 22)	Non-abdominal obesity (*n* = 24)
Age (years old)	0.360	35.27 ± 8.93	34.04 ± 9.68
Height (cm)	0.159	164.90 ± 10.72	160.71 ± 7.38
Weight (kg)	0.000*	81.47 ± 21.23	60.48 ± 12.66
BMI (kg/m^2^)	0.000*	29.71 ± 5.34	23.32 ± 4.20
Waist circumference (cm)	0.000*	98.83 ± 11.11	81.37 ± 11.30
Fat mass (%)	0.005*	31.93 ± 6.57	25.94 ± 7.05
Muscle mass (%)	0.010*	27.62 ± 5.41	31.40 ± 5.05
Visceral fat (%)	0.000*	12.32 ± 3.44	6.96 ± 2.91
Systolic blood pressure (mmHg)	0.085	129.77 ± 16.56	121.67 ± 12.73
Diastolic blood pressure (mmHg)	0.097	89.18 ± 9.46	84.75 ± 8.71
Energy (kcal)	0.296	2043.02 ± 438.46	1907.65 ± 430.54
Protein (g)	0.257	88.76 ± 23.11	80.67 ± 22.14
Fat (g)	0.531	81.73 ± 21.00	77.30 ± 25.97
Carbohydrate (g)	0.350	245.89 ± 58.55	226.18 ± 52.86
Fiber (g)	0.982	17.45 ± 4.55	16.95 ± 5.17
PUFA (g)	0.296	20.56 ± 7.40	18.25 ± 6.09

a Mann–Whitney U Test.

All data are in Mean ± SD, with *p*-value of <0.05 (indicated with star (*)) being significant. BMI, Body mass index; PUFA, Polyunsaturated fatty acids.

### 3.2 Heatmap analysis

**Figure 2:**
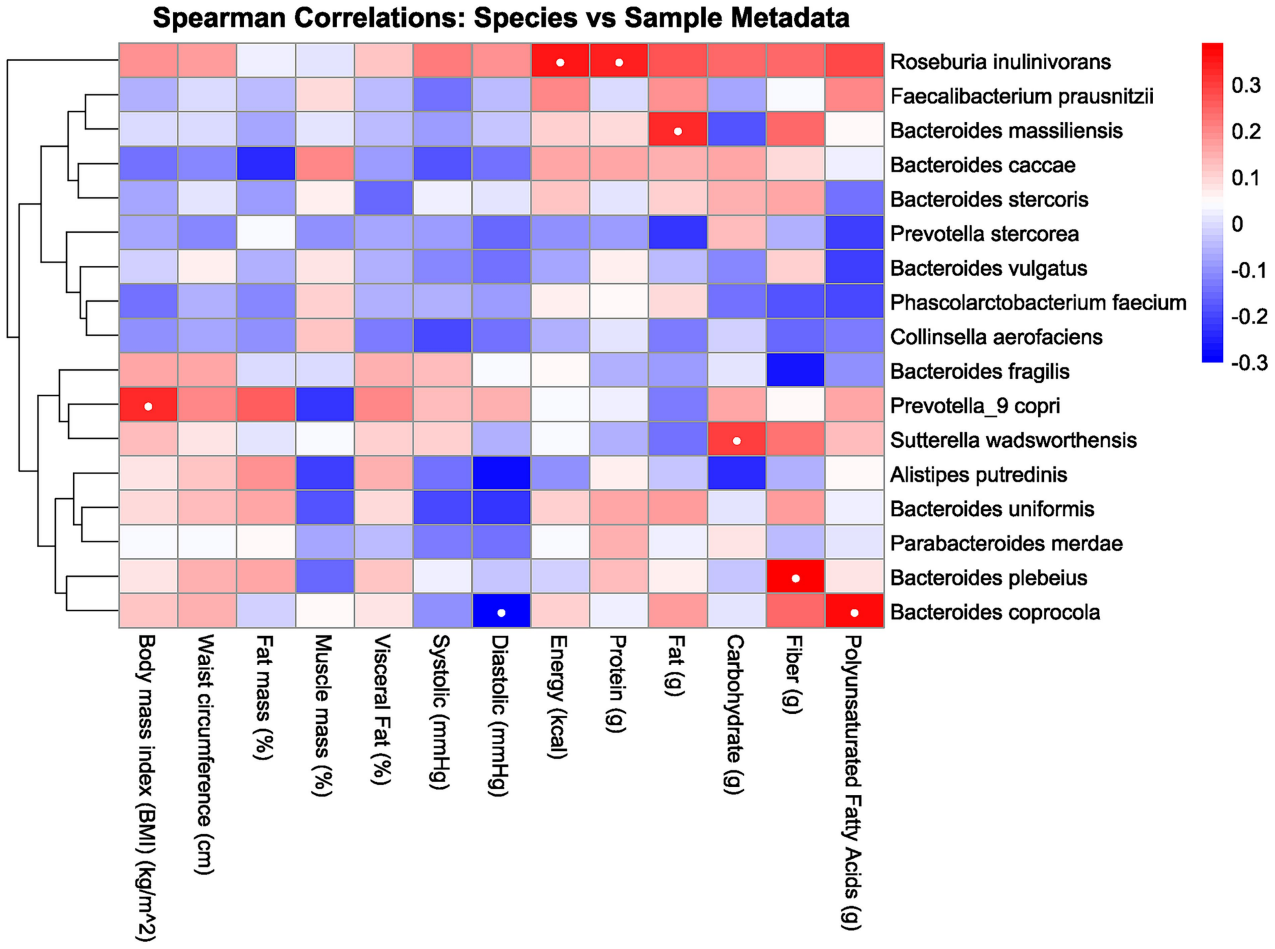
Heatmap correlation between the gut microbiota, body composition, and macronutrient intake. Red indicates a positive correlation, and blue indicates a negative correlation. The intensity of the color corresponds to the strength of the correlation, with deeper colors indicating stronger relationships. White dots inside the box indicate significance.

The heatmap analysis in Figure 2 reveals distinct correlations between human health parameters and gut microbiota composition, with more than 80% represented by the following taxa: *Roseburia inulinivorans, Faecalibacterium prausnitzii, Bacteroides massiliensis, Bacteroides caccae, Bacteroides stercoris, Prevotella stercorea, Bacteroides vulgatus, Phascolarctobacterium faecium, Collinsella aerofaciens, Bacteroides fragilis, Prevotella_9 copri, Sutterella wadsworthensis, Alistipes putredinis, Bacteroides uniformis, Parabacteroides merdae, Bacteroides plebeius,* and *Bacteroides coprocola*. In this analysis, significant correlations were observed between several species with biomarkers, with interpreted key details available in Table 2.

**Table 2:** Species-to-biomarker key highlights from Spearman correlations analysis.

Variable	Species	Correlation value	*p*-Value^a^
BMI	*Prevotella_9 copri*	0.322	0.029
VF*		0.206	0.169
Diastolic blood pressure	*Bacteroides coprocola*	−0.302	0.032
PUFA		0.364	0.013
Energy	*Roseburia inulinivorans*	0.349	0.018
Protein		0.334	0.023
Fat	*Bacteroides massiliensis*	0.326	0.027
Carbohydrate	*Sutterella wadsworthensis*	0.294	0.047
Fiber	*Bacteroides plebeius*	0.388	0.008

a Adjusted *p*-value.

*Indicating species-to-biomarker of interest. BMI, Body mass index; VF, Visceral fat; PUFA, Polyunsaturated fatty acid.

### 3.3 Alpha and beta diversity

Alpha diversity analysis using species richness and diversity indices showed no significant differences between abdominal obesity and non-abdominal obesity groups. Species numbers were similar (*p* = 0.9), and Shannon, Simpson, and Inverse Simpson indices showed no differences (*p* = 0.72, 0.5, 0.5), indicating similar microbial diversity between groups. Beta diversity analysis using NMDS and PCoA plots revealed no significant separation between groups, as supported by PERMANOVA results (R^2^ = 0.016, *p* = 0.953), indicating minimal compositional differences in gut microbiota.

## 4 Discussion

This study identified several significant correlations among various species and biomarkers, as demonstrated through heatmap analysis in Figure 2 and detailed in Table 2. There are several potential metabolic functions of the identified microbes according to several studies. *Prevotella_9 copri* is notably involved in amino acid and carbohydrate metabolism (25), while *Bacteroides coprocola* has shown a characteristic distribution of single-nucleotide polymorphisms (SNPs) in type 2 diabetes patients (26). *Roseburia inulinivorans* is recognized for its utilization of inulin and starch (27), and *Bacteroides massiliensis* also plays a role in carbohydrate metabolism (28). *Sutterella wadsworthensis* demonstrates potential binding to mucus and extracellular matrix proteins (29), and *Bacteroides plebeius* has been isolated from seaweed-eating Japanese individuals (30). From these identified microbes, several correlations were observed. For instance, BMI exhibited a positive correlation with *Prevotella_9 copri*, consistent with findings from a study examining this relationship in children (31). While waist circumference correlated positively with *Prevotella_9 copri* and *Bacteroides coprocola*, no prior studies have reported similar findings. These species are abundant in individuals consuming carbohydrate-rich diets, suggesting a potential link between these species and waist circumference via carbohydrate intake. However, our study found no significant correlation between these species and carbohydrate consumption. (25, 32, 33). Several studies have reported findings that differ from those of the present study. For instance, Asnicar et al. (34) identified an inverse correlation between *Prevotella_9 copri* and visceral fat, whereas this study observed a positive correlation. This is interesting because while there is a possible explanation of increasing PUFA serum level as the reason of negative correlation in one of the studies, in this study *Prevotella_9 copri* are having no significance with the PUFA serum level (34). Furthermore, there are few researches that explore the relationship between other species and biomarkers. This scarcity may be attributed to the limited exploration of species-biomarker correlation, especially in Indonesia, where dietary intake and other biomarkers differ from those in other regions.

Alpha diversity analysis showed no significant differences in species richness and diversity indices between groups, indicating maintained microbial biodiversity regardless of abdominal obesity status. Beta diversity analysis revealed no segregation of gut microbiota community structures between abdominal obesity and non-abdominal obesity groups, with NMDS and PCoA plots showing no clustering differences. These findings contrast with prior studies that demonstrated distinct clustering patterns between metabolically unhealthy individuals and healthy controls, suggesting obesity-linked microbial composition differences (35, 36). However, in this study, such clear distinctions were not observed. The lack of correlation between alpha and beta diversity may be explained by their different aspects: alpha diversity measures the richness and evenness within a single sample, whereas beta diversity measures the differences between samples. Study limitations include low sample size (*n* = 46) and cross-sectional design preventing causal inference. While taxonomic profiles were analyzed, microbial metabolite functions were not studied. Furthermore, other associated factors were not observed such as physical activity that showed the effects of the overall microbiota composition, limiting the generalizability (37), However, given the nature of this study as a pilot study, this work provides the groundwork for future microbiota research.

In conclusion, this study revealed a potential uniqueness in one of the gut microbiota, with *Prevotella_9 copri* showing an inverse correlation with visceral fat compared to findings from other studies conducted outside of Indonesia. These microbial profiles may serve as biomarkers for early detection and personalized interventions. However, the small sample size and cross-sectional design restrict the generalizability and causal inference. Future studies with larger sample sizes and comparisons across urban and rural locations, including coastal and highland regions, will be beneficial to validate these associations, enhance understanding of the underlying mechanisms, and advance microbiota-targeted strategies for managing abdominal obesity and its metabolic consequences.

## Data Availability

The original contributions presented in the study are included in the article/supplementary material; further inquiries can be directed to the corresponding author.
